# Two-dimensional shear wave elastography: a new tool for evaluating respiratory muscle stiffness in chronic obstructive pulmonary disease patients

**DOI:** 10.1186/s12890-022-02231-4

**Published:** 2022-11-24

**Authors:** Yongjian Chen, Jingyun Li, Bingtian Dong, Zhixing Zhu, Guorong Lyu

**Affiliations:** 1 Department of Ultrasound, the Second Affiliated Hospital of Fujian Medical University, No. 34 North Zhongshan Road, Licheng District, 362000 Quanzhou, Fujian China; 2 Quanzhou Medical College, No. 2 Anji Road, Luojiang District, 362000 Quanzhou, Fujian Province China; 3 Department of Pulmonary and Critical Care Medicine, the Second Affiliated Hospital of Fujian Medical University, No. 34 North Zhongshan Road, Licheng District, 362000 Quanzhou, Fujian China

**Keywords:** Chronic obstructive pulmonary disease, Two-dimensional shear wave elastography, Diaphragm stiffness, Intercostal muscle stiffness, Lung function

## Abstract

**Background:**

Impaired respiratory function caused by respiratory muscle dysfunction is one of the common consequences of chronic obstructive pulmonary disease (COPD). In this study, two-dimensional shear wave elastography (2D-SWE) was used to measure diaphragm stiffness (DS) and intercostal muscle stiffness (IMS) in patients with COPD; in addition, the value of 2D-SWE in evaluating respiratory function was determined.

**Methods:**

In total, 219 consecutive patients with COPD and 20 healthy adults were included. 2D-SWE was used to measure the DS and IMS, and lung function was also measured. The correlation between respiratory muscle stiffness and lung function and the differences in respiratory muscle stiffness in COPD patients with different severities were analysed.

**Results:**

2D-SWE measurements of the DS and IMS presented with high repeatability and consistency, with ICCs of 0.756 and 0.876, respectively, and average differences between physicians of 0.10 ± 1.61 and 0.07 ± 1.65, respectively. In patients with COPD, the DS and IMS increased with disease severity (F_1_ = 224.50, F_2_ = 84.63, *P* < 0.001). In patients with COPD, the correlation with the forced expiratory volume in one second (FEV_1_)/forced vital capacity (FVC), predicted FEV_1_% value, residual volume (RV), total lung capacity (TLC), RV/TLC, functional residual capacity (FRC) and inspiratory capacity (IC) of DS (r_1_=-0.81, r_2_=-0.63, r_3_ = 0.65, r_4_ = 0.54, r_5_ = 0.60, r_6_ = 0.72 and r_7_=-0.41, respectively; *P* < 0.001) was stronger than that of IMS (r_1_=-0.76, r_2_=-0.57, r_3_ = 0.57, r_4_ = 0.47, r_5_ = 0.48, r_6_ = 0.60 and r_7_=-0.33, respectively; *P* < 0.001).

**Conclusion:**

2D-SWE has potential for use in evaluating DS and IMS. A specific correlation was observed between respiratory muscle stiffness and lung function. With the worsening of the severity of COPD and the progression of lung function impairment, the DS and IMS gradually increased.

## Background

Chronic obstructive pulmonary disease (COPD) is a high-incidence chronic respiratory disease characterized by incomplete reversible airflow limitation and progressive development [1]. The diaphragm is the most important respiratory muscle; thus, when a COPD patient develops diaphragmatic dysfunction, external respiratory dysfunction, alveolar hypoventilation, dyspnoea, hypercapnia and even premature death may occur [2, 3]. Diaphragm dysfunction leads to patients being unable to tolerate general daily activities; moreover, diaphragm fatigue causes long-term low oxygen and carbon dioxide retention, and the most severe cases can lead to respiratory failure that requires mechanical ventilation [4, 5]. Diaphragmatic dysfunction is associated with an increased risk of hospitalization caused by acute COPD aggravation, which significantly reduces the quality of daily life and increases the difficulty of treatment during the acute exacerbation period. The evaluation of diaphragm impairment plays an important role in disease assessments, targeted rehabilitation therapy and pulmonary physiotherapy [6, 7]. The diaphragm function of COPD patients is closely related to lung function; therefore, the identification of a simpler method of evaluating the diaphragm function of COPD patients has important clinical significance.

At present, researchers use ultrasound technology to noninvasively evaluate diaphragm function, and the primary focus of ultrasound is on the degree of diaphragm movement and contraction amplitude [8, 9]. Ultrasound technology supplements respiratory muscle strength testing but cannot replace it [10]. Therefore, the development of a method that can better evaluate the function of the diaphragm has become a new research hotspot in ultrasound medicine. Shear wave elastography (SWE) monitors the propagation of shear waves in tissue and utilizes an acoustic radiation force pulse sequence to generate shear waves, which propagate perpendicular to the ultrasound beam, thus causing transient displacements. The shear wave velocity (SWV) varies with the mechanical properties of tissues, such as stiffness. Stiffness is subsequently quantified by the shear modulus, which is calculated through shear wave propagation. SWV and shear modulus can be expressed by the following formula: µ = ρv^2^ (µ represents shear modulus, and µ expressed in kPa; ρ represents the density of the tissue, which is equal to 1000 kg/m^3^ in the human body; and v represents SWV) [11, 12]. SWE energizes the elasticity of muscles to obtain biomechanical information, which can be used for the diagnosis of degenerative diseases and the evaluation of treatment efficacy and disease severity [13, 14]. SWE is considered to be a suitable ultrasound elastography technique for evaluating the muscle system and may represent a promising area for the advancement of ultrasound imaging technology [15]. Previous research by our group showed that the use of virtual touch tissue imaging quantification (VTIQ) to measure diaphragm stiffness can effectively assess the severity of COPD [16]. However, compared with two-dimensional shear wave elastography (2D-SWE), VTIQ has a larger sampling frame and multipoint sampling; therefore, its consistency and repeatability are poor, and its clinical application is limited. At present, few studies have focused on 2D-SWE technology for evaluating diaphragm function. Bachasson found that the 2D-SWE measurement of the diaphragm elastic modulus can effectively reflect the transdiaphragmatic pressure [17]. In addition, Chino used 2D-SWE to evaluate the diaphragm elastic modulus under maximum oral inspiratory pressure and showed that this parameter increases with increasing pressure [18]. 2D-SWE provides a new opportunity for the non-invasive evaluation of diaphragm function. The chest wall contractility of COPD patients has been shown to be weakened [19]. The ultrasound measurement of parasternal intercostal muscle thickness and echo score are closely related to the forced expiratory volume in one second (FEV_1_), and the correlation is stronger than that of CT measurements [20]. However, the elasticity of the parasternal intercostal muscles has not been previously reported. The study by Flatres [21] showed that SWE can quantify the muscle mass of patients with myasthenia gravis, and it has high repeatability and accuracy. This finding inspired us to investigate whether SWE may also be suitable for evaluating intercostal muscle function in COPD patients and whether SWE can jointly predict the severity of lung function impairment in COPD patients to a certain extent, which is worthy of further study.

## Methods

### Participants

A total of 219 COPD patients who were seen in the ultrasound department of our hospital between June 2020 and May 2022 were selected, and 20 healthy adults who were seen during the same time period were selected as the control group. The age of the control group was essentially in the same range as that of the experimental group. According to the 2020 edition of the GOLD “Global Initiative for Chronic Obstructive Pulmonary Disease”, the diagnosis of COPD was based on clinical symptoms, signs, chest X-ray or CT findings, lung function parameters and other examination findings. The exclusion criteria for the COPD group were as follows: (1) history of respiratory disease; (2) history of pleural disease and peritoneal disease; (3) history of thoracic or abdominal trauma or surgery; (4) chronic metabolic disease; (5) history of using drugs that affect the structure of skeletal muscle tissue; (6) unsuitability to cooperate with the study; and (7) history of respiratory infection in the previous month before the study. All of the healthy individuals were subject to the same exclusion criteria; furthermore, they met pulmonary function test results showing FEV_1_/FVC ≥ 70% and FEV_1_ ≥ 80% of the predicted value. All of the subjects underwent chest radiography within 2 weeks. Based on the 2020 edition of the GOLD “Global Initiative for Chronic Obstructive Pulmonary Disease”, COPD patients were divided into mild, moderate, severe and very severe groups (Table 1). General data on the subjects, including sex, age and body mass index, were collected, and all of the participants completed the modified Medical Research Council (mMRC) Dyspnoea Scale, COPD Assessment Test (CAT), lung function tests and SWE. This study was approved by the hospital ethics committee, and all of the subjects agreed to undergo the examinations and provided signed informed consent.

**Table 1:** Classification of COPD

Classification	FEV_1_/FVC	FEV_1_% predicted
Mild	< 70%	FEV_1_ ≥ 80% predicted
Moderate	< 70%	50%≤FEV_1_< 80% predicted
Severe	< 70%	30%≤FEV_1_< 50% predicted
Very Severe	< 70%	FEV_1_< 30% predicted

*COPD* chronic obstructive pulmonary disease, *FEV*_*1*_ forced expiratory volume in one second, *FVC *forced vital capacity

### Instruments and methods

**Fig. 1:**
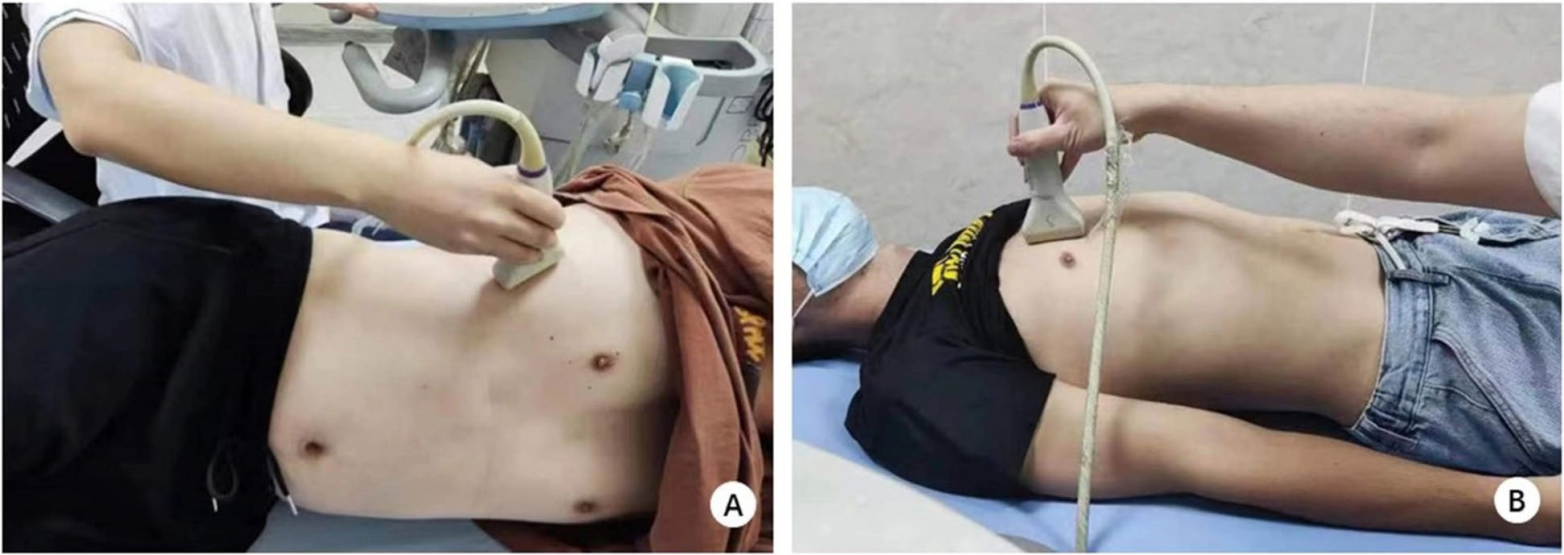
2D-SWE measurement of respiratory muscle position. **A** Diaphragm measurement position. **B** Intercostal muscle measurement position

**Fig. 2:**
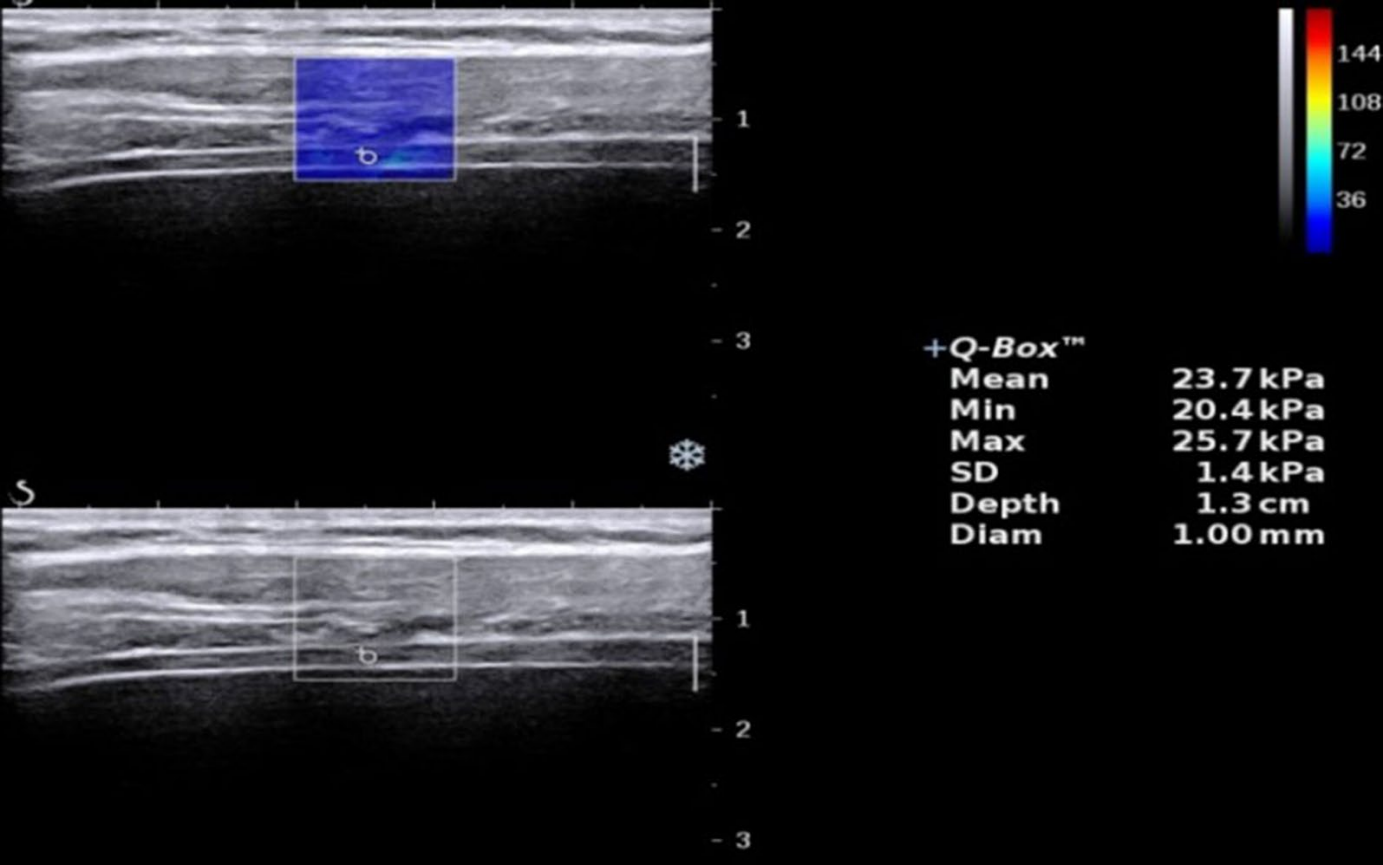
2D-SWE measurement of the elastic modulus of the diaphragm in the control group

**Fig. 3:**
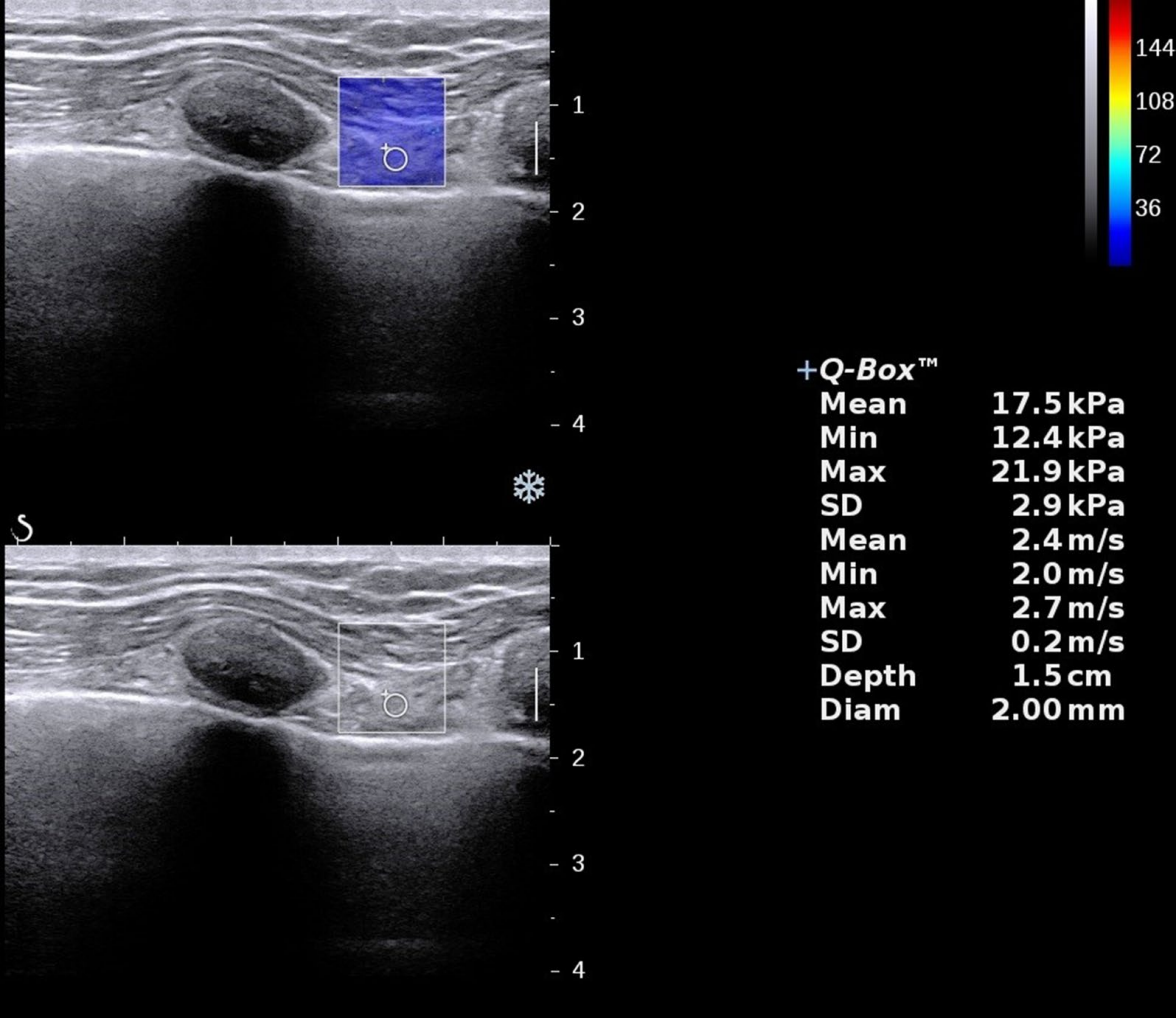
2D-SWE measurement of the elastic modulus of intercostal muscles in the control group

The high-resolution ultrasonic diagnostic apparatus (Aixplorer, Supersonic Imagine, Provence, France) was used, with a 4–15 MHz frequency linear array probe. Each subject was instructed to remain in the left lying position. The physician placed the probe under the rib between the anterior to mid-axillary line and approximately the 6th to 12th intercostal space to allow for the probe to display the diaphragm to the greatest extent (Fig. 1). The gain was adjusted, and the depth was checked. After the image of the right diaphragmatic rib of the examinee became stable, the SWE mode was switched, and the elastic range was adjusted to 0-160 kPa. When the subject was calmly holding their breath at the end of inhalation, the elastic modulus of the diaphragm and intercostal muscles in the ROI (region of interest) was measured by using Q-BOX. When measuring the diaphragm, a circular area with a diameter of 1 mm was selected as the ROI. The elastic modulus of the diaphragm was measured three times, and the average value was taken (Fig. 2). The subject was instructed to move to a supine position, and the physician placed the probe on the intercostal muscles of the front chest wall to obtain images of the intercostal muscles (Fig. 1). The subject was then asked to hold his or her breath at the end of inhalation. After the image became stable, the SWE mode was turned on, and the elastic modulus was measured. The ROI was set to a circle with a diameter of 2 mm, and the average was taken after 3 measurements (Fig. 3). Similar to the measurement method of An [22], the diaphragm excursion (DE) and diaphragm thickening fraction (TF) were measured at tidal inspiration and at maximal deep inspiration. Pulmonary function tests were performed in accordance with the standards of the European Respiratory Society [23], and the FEV_1_, forced vital capacity (FVC), FEV_1_/FVC, inspiratory capacity (IC), functional residual capacity (FRC), residual volume (RV), total lung capacity (TLC) and RV/TLC were recorded.

### Statistical analysis

Normally distributed measurement data are expressed as the mean ± SD, and SPSS (version 19.0; SPSS) was used for all of the analyses. The inspection level α was taken as 0.05. Nonnormally distributed data are presented as the median [IQR] or n (%), as appropriate. The intraclass correlation coefficient (ICC) and a Bland–Altman diagram were used to analyse the repeatability and consistency of 2D-SWE measurements of the diaphragm and intercostal elastic modulus. The relationship between the diaphragm elastic modulus, intercostal muscle elastic modulus and lung function was determined via the Pearson correlation analysis. The mild, moderate, severe, very severe and healthy control groups that were normally distributed were evaluated by using one-way ANOVA when the variance was homogeneous, and the mean differences between the groups were compared with the least significant difference LSD-t test. The data were evaluated via the Welch’s test when the variance was uneven, and the mean differences between the groups were compared via the Tamhane’s T2 test. Nonnormally distributed data and data with uneven variance were compared by using nonparametric tests (the Kolmogorov–Smirnov test).

## Results

### Basic clinical measurements and lung function

Table 2 compares the basic clinical measurement data and pulmonary function data between patients with different severities of COPD and healthy controls. Sex, age, smoking status and BMI were similar among the groups. However, compared with the healthy control group, individuals in the COPD group had a lower FEV_1_% predicted value, FEV_1_/FVC, IC, DE at tidal inspiration, DE at maximal deep inspiration, TF at tidal inspiration and TF at maximal deep inspiration, and these indicators continued to decrease with increased severity of the disease. Individuals in the COPD group had a higher smoking frequency, CAT score, mMRC score, RV, RV/TLC and FRC, and these indicators continued to increase with increased severity of the disease.

**Table 2:** Clinical characteristics of the participants with different severities of COPD

Characteristics	Control (*n* = 20)	Mild COPD (*n* = 82)	Moderate COPD (*n* = 52)	Severe COPD (*n* = 46)	Very severe COPD (*n* = 39)
Age (years)	66.30 (9.65)	68.94 (8.21)	64.44 (9.69)	67.76 (8.00)	65.38 (9.52)
Male participants (%)	18 (90%)	72 (87%)	48 (92%)	40 (87%)	39 (100%)
Body mass index (kg/m^2^)	20.54 (2.08)	20.68 (1.93)	19.83(1.62)	20.87 (2.74)	21.47 (3.70)
Smoking, n (%)					
Never	15 (75%)	14 (17%) ^a^	2 (4%) ^a^	0 (0%) ^a^	0 (0%) ^a^
Ex-smoker	2 (10%)	18 (22%)	4 (8%)	4 (9%)	2 (5%)
Current smoker	3 (15%)	50 (61%) ^a^	46 (88%) ^a^	42 (91%) ^a^	37 (95%) ^a^
Smoking (pack-years)	0 [0-11.25]	35 [18.75-40] ^a^	35 [30-45.75] ^ab^	45 [30–50] ^ab^	35 [35–50] ^ab^
GOLD group, n (%)					
A		16 (20%)	0 (0%)	0 (0%)	0 (0%)
B		22 (26%)	26 (50%)	16 (35%)	10 (26%)
C		8 (10%)	0 (0%)	0 (%)	0 (0%)
D		36 (44%)	26 (50%)	30 (65%)	29 (74%)
mMRC score	0 [0–0]	1 [0–1] ^a^	1.5 [1–2] ^ab^	2 [2–3] ^abc^	3 [2–4] ^abcd^
CAT score	5.50 [0.25-8]	13 [9–18] ^a^	22 [18-27.75] ^ab^	32 [29–34] ^abc^	34 [32–37] ^abcd^
FEV_1_% predicted value	89.90 (3.63)	87.72 (5.20)	64.56 (8.51) ^ab^	39.89 (5.24) ^abc^	20.85 (5.66) ^abcd^
FEV_1_/FVC	80.50 (2.57)	59.17 (5.52) ^a^	55.29 (5.04) ^ab^	46.65(6.74) ^abc^	36.15(7.03) ^abcd^
RV (L)	1.86 [1.66–1.98]	2.15 [1.84–2.34] ^a^	2.38 [2.16–2.59] ^ab^	2.96 [2.71–3.27] ^abc^	3.36 [2.86–3.77] ^abc^
TLC (L)	5.70 (0.30)	5.46 (0.35) ^a^	5.79 (0.42) ^b^	6.19 (0.39) ^abc^	6.33 (0.50) ^abc^
RV/TLC	0.33 [0.30–0.35]	0.38 [0.35–0.44] ^a^	0.42 [0.38–0.45] ^ab^	0.49 [0.45–0.53] ^abc^	0.54 [0.46–0.59] ^abc^
FRC (L)	2.68 (0.28)	3.11 (0.37) ^a^	3.35 (0.44) ^ab^	4.20 (0.39) ^abc^	4.66 (0.36) ^abcd^
IC (L)	3.00 [2.90–3.20]	2.30 [2.00-2.70] ^a^	2.50 [2.00-2.80] ^a^	2.00 [1.57–2.33] ^abc^	1.70 [1.40–2.10] ^abc^
DE at tidal inspiration (mm)	21.90 [18.65–27.13]	23.90 [21.25–26.23]	20.60 [19.23–22.55]^b^	17.05 [15.18–18.10] ^abc^	16.40 [13.60–18.80] ^abc^
DE at maximal deep inspiration (mm)	60.10 [55.18–76.33]	36.45 [23.58–49.60] ^a^	34.45 [29.55–40.08] ^a^	37.30 [33.85–39.13] ^a^	33.10 [29.80–40.30] ^a^
TF at tidal inspiration	1.30 [1.00-1.60]	0.67 [0.53–0.78] ^a^	0.54 [0.48–0.68] ^ab^	0.35 [0.22–0.45] ^abc^	0.12 [0.02–0.41] ^abc^
TF at maximal deep inspiration	1.92 [1.40–2.59]	1.69 [1.53–1.98]	1.17 [0.90–1.50] ^ab^	0.89 [0.46–1.18] ^abc^	0.73 [0.19–1.08] ^abc^

All data are presented as either n (%) for categorical variables or median [IQR] and mean (SD) for continuous variables

*COPD* chronic obstructive pulmonary disease, *GOLD *Global Initiative for Chronic Obstructive Lung Disease, *mMRC* modified Medical Research Council, *CAT *COPD Assessment Test, *FEV*_*1*_ forced expiratory volume in one second, *FVC* forced vital capacity, *RV* residual volume, *TLC* total lung capacity, *FRC* functional residual capacity, *IC* inspiratory capacity, *DE* diaphragm excursion, *TF* thickening fraction

^a^compared with the healthy control group, *P* < 0.05; ^b^compared with the mild COPD group, *P* < 0.05; ^c^compared with the moderate COPD group, *P* < 0.05; ^d^ compared with the severe COPD group, *P* < 0.05

### Comparison of the consistency and repeatabilityof the diaphragm elastic modulus and intercostal elastic modulus measured via the 2D-SWE technique

- Reliability of diaphragm and intercostal muscle elastic modulus measurements performed by the same physician by using 2D-SWE technology.

**Fig. 4:**
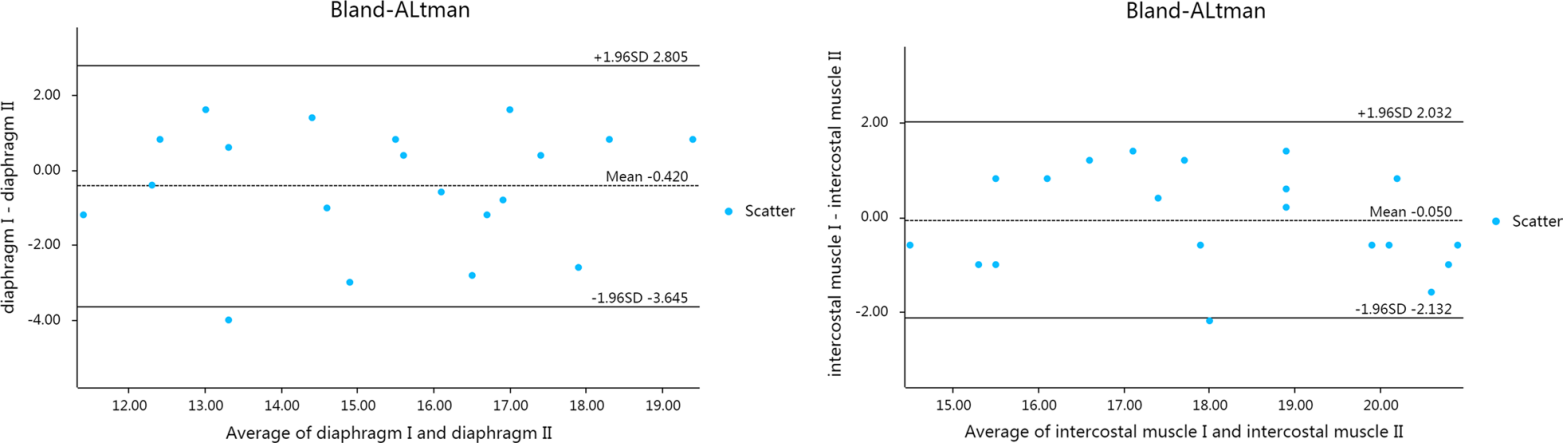
Comparison of Bland–Altman analysis graphs for the diaphragm and intercostal muscle elastic modulus measurements performed by the same physician in the control group by using 2D-SWE

The same physician used 2D-SWE technology to measure the elastic modulus of the diaphragm and intercostal muscle over two measurements, and good repeatability was observed (Fig. 4; Table 3). 5% (1/20) and 5% (1/20) of the differences between the two diaphragm elastic modulus and intercostal elastic modulus measurements were outside of the consistency limit, respectively (Fig. 4). The mean of the difference was close to 0, and most of the other points were close to the average data; therefore, these differences can be accepted in practical clinical use. Moreover, the intragroup correlation coefficients (ICCs) of the two sets of data measured by the same physician were high (Table 3).

**Table 3:** Reliability of the elastic modulus of the diaphragm and intercostal muscle measurements performed by the same physician by using 2D-SWE technology

	Consistency analysis		Repeatability analysis	
	Mean difference between the two measurements	95% agreement limit	ICC	95% CI
Elastic modulus of diaphragm	-0.42 ± 1.65	-3.65 ~ 2.81	0.756	0.482 ~ 0.896
Elastic modulus of intercostal muscle	-0.05 ± 1.06	-2.13 ~ 2.03	0.876	0.712 ~ 0.949

*2D-SWE* two-dimensional shear wave elastography, *ICC *intraclass correlation coefficient, *CI* consistency index

- Reliability of diaphragm and intercostal muscle elastic modulus measurements made by different physicians by using 2D-SWE technology.

**Fig. 5:**
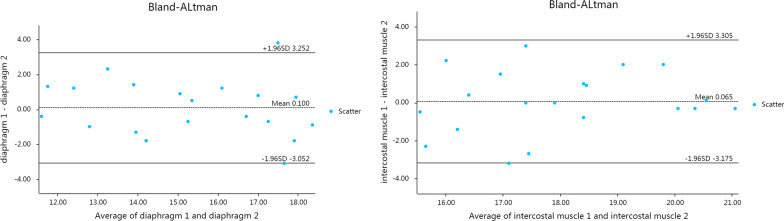
Comparison of Bland–Altman analysis graphs for the diaphragm and intercostal muscle elastic modulus measurements performed by different physicians in the control group by using 2D-SWE

Different physicians used 2D-SWE technology to measure the elastic modulus of the diaphragm and intercostal muscles with high consistency (Fig. 5; Table 4). 5% (1/20) and 0% (0/20) of the differences between the diaphragm elastic modulus and intercostal elastic modulus measurements made by different physicians were outside of the consistency limit, respectively (Fig. 5). The mean difference was close to 0, and most of the other points were around the average data; therefore, these differences can be accepted in practical clinical use. The ICCs of the diaphragm and intercostal muscle elastic modulus measurements made by different physicians were both modest (Table 4).

**Table 4:** Reliability of the elastic modulus of the diaphragm and intercostal muscle measurements performed by different physicians by using 2D-SWE technology

	Consistency analysis		Repeatability analysis	
	Mean difference between physicians	95% agreement limit	ICC	95% CI
Elastic modulus of diaphragm	0.10 ± 1.61	-3.05 ~ 3.25	0.775	0.511 ~ 0.905
Elastic modulus of intercostal muscle	0.07 ± 1.65	-3.18 ~ 3.31	0.628	0.260 ~ 0.835

*2D-SWE* two-dimensional shear wave elastography, *ICC* intraclass correlation coefficient, *CI* consistency index

### Analysis of the diaphragm and intercostal muscle elastic modulus measurements in patients with different severities of COPD

**Fig. 6:**
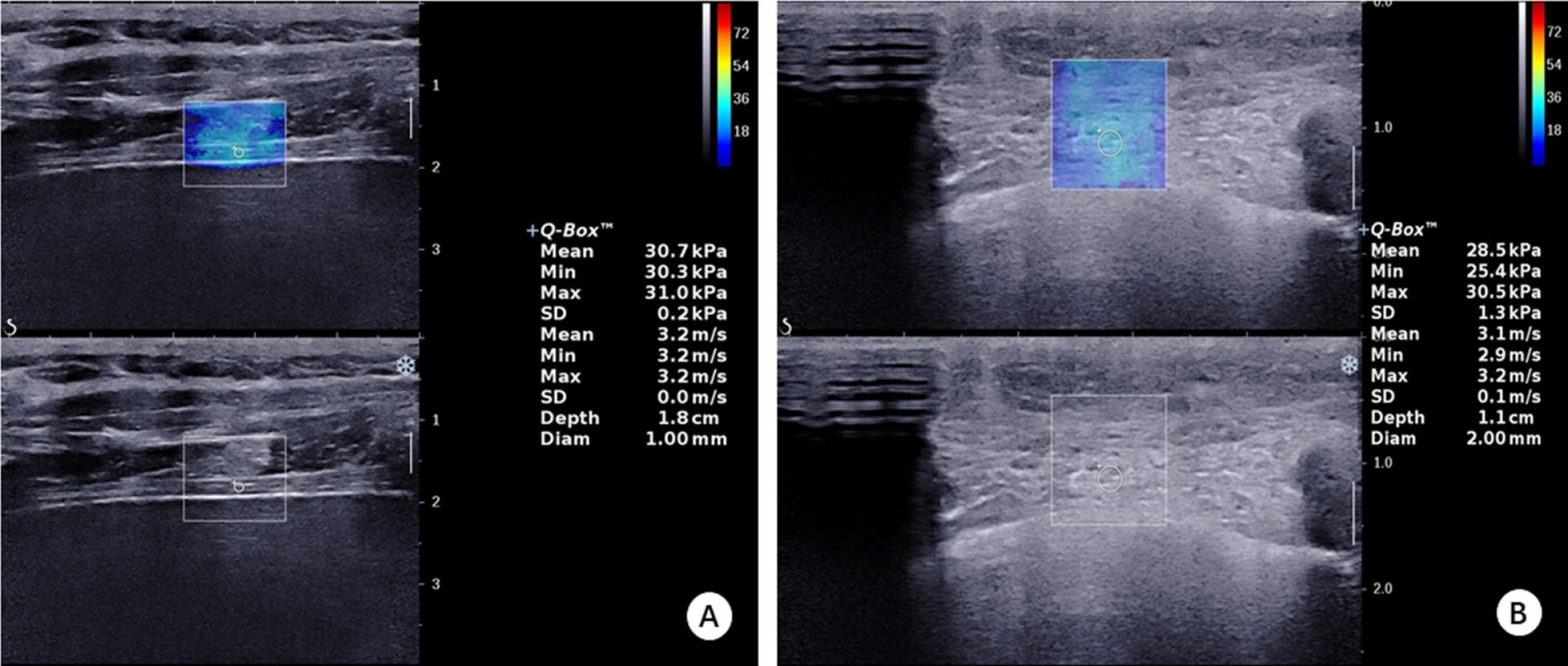
2D-SWE measurement of the elastic modulus of respiratory muscle in patients with COPD. **A** Elastic modulus of the diaphragm in patients with COPD. **B** Elastic modulus of the intercostal muscles in patients with COPD

The differences in elastic modulus measurements between the healthy control group and patients with different severities of COPD were compared. Significant differences in the diaphragm elastic modulus were not observed between the severe COPD group and the very severe COPD group. As the severity of COPD increased in each of the remaining two groups, the elastic modulus of the diaphragm and intercostal muscles increased (Fig. 6; Table 5).

**Table 5:** Differences in the elastic modulus of the diaphragm and intercostal muscles in patients with different severities of COPD (kPa, *X*±*S*)

	Elastic modulus of diaphragm	Elastic modulus of intercostal muscle
Control (*n* = 20)	15.35 ± 2.22	18.04 ± 2.02
Mild COPD (*n* = 82)	23.78 ± 4.49^a^	25.39 ± 5.60^a^
Moderate COPD (*n* = 52)	33.49 ± 6.11^ab^	33.95 ± 7.68^ab^
Severe COPD (*n* = 46)	43.12 ± 5.89^abc^	40.32 ± 9.22^abc^
Very severe COPD (*n* = 39)	44.38 ± 4.47^abc^	46.25 ± 9.45^abcd^
*F* value	224.50	84.63
*P* value	< 0.001	< 0.001

*COPD* chronic obstructive pulmonary disease

^a^compared with the healthy control group, *P* < 0.05; ^b^compared with the mild COPD group, *P* < 0.05; ^c^compared with the moderate COPD group, *P* < 0.05, ^d^compared with the severe COPD group, *P* < 0.05

### Correlation analysis of the diaphragm and intercostal muscle elastic modulus and lung function in patients with COPD

For patients with COPD, the diaphragm elastic modulus was negatively correlated with the predicted FEV_1_/FVC and FEV_1_%, and the correlation was stronger than that of the intercostal muscle elastic modulus and the predicted FEV_1_/FVC and FEV_1_%. Other commonly used ultrasound indicators, such as TF and DE, had weaker correlations with the predicted FEV_1_/FVC and FEV_1_% values than the elastic modulus of the diaphragm (Table 6). The elastic moduli of the diaphragm and intercostal muscles were correlated with all lung volumes, and TF and DE were correlated with most lung volume indices (Table 7). In addition, the diaphragm elastic modulus was positively correlated with the intercostal muscle elastic modulus (*r* = 0.56, *P* < 0.001).

**Table 6:** Correlation between the ultrasonic indicators and lung ventilation function in COPD

	FEV_1_% predicted value		FEV_1_/FVC	
	r	*P*	r	*P*
Elastic modulus of diaphragm	-0.81	< 0.001	-0.63	< 0.001
Elastic modulus of intercostal muscle	-0.76	< 0.001	-0.57	< 0.001
DE at tidal inspiration	0.55	< 0.001	0.47	< 0.001
DE at maximal deep inspiration	0.10	0.138	0.23	0.001
TF at tidal inspiration	0.56	< 0.001	0.48	< 0.001
TF at maximal deep inspiration	0.58	< 0.001	0.60	< 0.001

*COPD* chronic obstructive pulmonary disease, *FEV*_*1*_ forced expiratory volume in one second, *FVC *forced vital capacity, *DE *diaphragm excursion, *TF *thickening fraction

**Table 7:** Correlation between the ultrasonic indicators and lung volume in COPD

	RV		TLC		RV/TLC		FRC		IC	
	r	*P*	r	*P*	r	*P*	r	*P*	r	*P*
Elastic modulus of diaphragm	0.65	< 0.001	0.54	< 0.001	0.60	< 0.001	0.72	< 0.001	-0.41	< 0.001
Elastic modulus of intercostal muscle	0.54	< 0.001	0.47	< 0.001	0.48	0.001	0.60	< 0.001	-0.33	< 0.001
DE at tidal inspiration	-0.54	< 0.001	-0.37	< 0.001	-0.53	< 0.001	-0.48	< 0.001	0.26	< 0.001
DE at maximal deep inspiration	-0.02	0.771	-0.06	0.406	-0.01	0.913	-0.12	0.076	0.10	0.140
TF at tidal inspiration	-0.52	< 0.001	-0.26	0.019	-0.58	< 0.001	-0.48	< 0.001	0.37	< 0.001
TF at maximal deep inspiration	-0.58	< 0.001	-0.45	< 0.001	-0.55	< 0.001	-0.56	< 0.001	0.29	< 0.001

*COPD* chronic obstructive pulmonary disease, *RV *residual volume, *TLC *total lung capacity, *FRC *functional residual capacity, *IC *inspiratory capacity, *DE* diaphragm excursion, *TF* thickening fraction

## Discussion

Compared with ordinary ultrasound examination, ultrasound elastography can obtain biomechanical information. At present, only Xu and Şendur have studied diaphragmatic shear wave elastography in patients with COPD [24, 25]. Compared with the extensive research performed on the function of the diaphragm, research on the function of the intercostal muscles mainly includes electromyography and ordinary two-dimensional ultrasound [26], and the use of elastography to assess intercostal muscles is limited to diseases, such as idiopathic scoliosis in adolescents [27]. The results of this study showed that it is feasible to use 2D-SWE to detect the stiffness of the diaphragm and intercostal muscles of COPD patients, and the consistency and repeatability of its measurements were modest. Therefore, 2D-SWE has certain research prospects in detecting the stiffness of the diaphragm and intercostal muscles of COPD patients.

Our study also observed increases in diaphragm and intercostal muscle stiffness in patients with COPD, which supports the review by Xu [24]. Previous studies have shown that COPD can lead to an increase in oxidative metabolism [28] and can induce oxidative stress [29]. To increase resistance to respiratory muscle fatigue, the proportion of type I fibres (slow-contracting fibres) is increased [30, 31], which is often accompanied by myolysis, sclerosis and myofibril contracture [32], which can cause diaphragm remodelling and can increase diaphragm stiffness.

With the increasing severity of COPD, RV and TLC were gradually increased; additionally, RV/TLC also increased, and the hyperinflation intensified by degrees [33, 34]. The increase in FRC reflects the scenario that COPD patients may have decreased lung elasticity, and the balance between thoracic and lung tissue elasticity is destroyed [35]. Reduced IC suggests decreased maximal ventilatory potential. With the exacerbation of dynamic hyperinflation and air retention, pulmonary function is impaired, and FEV_1_/FVC and FEV_1_% values can decrease. The increased airway resistance and dynamic lung hyperinflation, which are characteristics of COPD, make the respiratory muscles work chronically against an increased load and can lead to the changed length-strength relationship and their remodelling [36]; subsequently, respiratory muscle dysfunction may develop. In our study, DE at tidal inspiration and TF that gradually decreased with increased COPD severity are evidence of progressive diaphragm dysfunction. Our results show that more severe degree of COPD corresponds to a more dysfunctional respiratory muscle; hence, the muscle will become stiffer.

This study also found that the correlation between diaphragm stiffness and lung function in patients with COPD was stronger than that between diaphragm stiffness and intercostal muscle stiffness. The main power of the breathing pump comes from the respiratory muscles, of which the diaphragm is the most important load-carrying muscle of inspiratory function (approximately 70%) [37], with the intercostal muscles and other respiratory muscles contributing relatively little effect. In general, an increase in diaphragm stiffness has a greater impact on lung function than an increase in intercostal muscle stiffness. Therefore, for patients with COPD, the correlation between diaphragm stiffness and lung function is stronger than that between diaphragm stiffness and intercostal muscle stiffness.

In clinical work, patients with acute exacerbations of COPD mainly have thoracic activity above the level of normal breathing, as well as abnormal breathing movements (including mostly shallow and rapid breathing) [38]. Patients with shallow and rapid breathing have poor coordination, which makes it more difficult to measure the diaphragm. However, the measurement of the intercostal muscles is less affected by breathing. In addition, DS was positively correlated with the IMS in our study. Therefore, it is possible to choose the IMS to evaluate the respiratory function of COPD patients with acute exacerbations (instead of the DS).

There were still several limitations to our study. First, we did not measure trans-diaphragmatic pressure or mouth pressure, which can directly assess diaphragm function. As most COPD patients in this study only needed conservative treatment with drugs, they did not meet the criteria for the collection of clinical invasive diaphragm function tests. In addition, the use of SWE for the diaphragm is operator-dependent and position dependent, and the interobserver differences that were observed in SWE measurements should be modified in the future for better applications. Finally, our groups lacked longitudinal measurements to compare with other studies [39], and our team is also planning to perform a clinical study on longitudinal measurements in the future.

An understanding of the role of SWE in the broader context of respiratory muscle dysfunction is an ongoing challenge. SWE may represent a promising area for the advancement of the assessment of respiratory muscle dysfunction. The correlational research should not be limited to COPD, and many events that may cause diaphragmatic dysfunction (such as Guillain‒Barre syndrome, myasthenia gravis, postpolio syndrome, mechanical ventilation and other factors) are worthy of further investigations by relevant scholars. Finally, as peripheral muscle dysfunction is a common complication in patients with COPD [40], ultrasound assessment of peripheral muscle function (particularly the quadriceps muscle) could be of interest [41]. SWE, which is a relatively newer imaging technique that allows for the quantitative measurement of the biomechanical properties of tissues, may play a new role in the peripheral muscle function assessment of COPD.

In summary, 2D-SWE can be used to assess the changes in the elastic modulus of the respiratory muscles of patients with different severities of COPD; additionally, combined with the two-dimensional ultrasound examination of diaphragm movement, contraction amplitude and other items, this technology can quantitatively evaluate respiratory muscle function.

## Conclusion

This clinical study showed that 2D-SWE can be used to assess the stiffness of the diaphragm and intercostal muscles of COPD patients, both of which are related to the lung functions of patients. In the progression of COPD, lung function impairment can develop, and the DS and IMS can gradually increase.

## Data Availability

The datasets generated and analyzed during the current study are not publicly due to privacy restrictions but available from the corresponding author upon reasonable request.

## Abbreviations

COPD: chronic obstructive pulmonary disease
2D-SWE: two-dimensional shear wave elastography
DS: diaphragm stiffness
IMS: intercostal muscle stiffness
SWE: shear wave elastography
SWV: shear wave velocity
VTIQ: virtual touch tissue imaging quantification
FEV_1_: forced expiratory volume in one second
GOLD: Global Initiative for Chronic Obstructive Lung Disease
mMRC: modified Medical Research Council
CAT: COPD Assessment Test
ROI: region of interest
DE: diaphragm excursion
TF: thickening fraction
FVC: forced vital capacity
IC: inspiratory capacity
FRC: functional residual capacity
RV: residual volume
TLC: total lung capacity
ICC: intraclass correlation coefficient
LSD: least significant difference
